# The Voluntary Hospitals Report

**Published:** 1923-08

**Authors:** 


### RECOVERY OF THE VOLUNTARY HOSPITALS.

### SIR NAPIER BURNETT'S REPORT.

SIR NAPIER BURNETT, Director of Hospital Services, Joint Council of the Order of St. John and the British Red Cross Society, has issued his fourth Annual Report on the Voluntary Hospitals of Great Britain, excluding London, which is under the supervision of King Edward's Fund (19 Berkeley Street, 1s.). Always a document of great importance, it is this year for two reasons of especial interest.

### Some Striking Figures.

"Firstly," writes Sir Arthur Stanley in his introductory note, "it shows the wonderful improvement in the finances of the hospitals, and, secondly, it refers to the work carried out in the hospital laboratories :—

"The figures relating to the financial position of the hospitals are very striking. They show that the ordinary income of the 666 hospitals under review failed to meet the ordinary expenditure by only £74,978, whereas, the deficit in the previous year was no less than £419,138. It is true that this wonderful improvement is mainly due to the decrease in ordinary expenditure, but Sir Napier is undoubtedly right in attributing the increase in ordinary income (£81,634 in England alone) to a deepening interest of the public in the voluntary hospitals movement. Four years ago—or even less—there were many of those engaged in hospital management who cordially disliked the idea of State or rate assistance, but thought one or the other inevitable. The figures set out in this report justify the hopes entertained, on the other hand, by those who believed that the voluntary system was founded on something more than the mere transitory needs of one section of the population. From every quarter help has come, and it is a most encouraging sign that there has been no falling off in the amounts received as annual subscriptions. In these days of high taxation it is obviously impossible to expect the large gifts such as the hospitals used to receive from generous benefactors before the War, but a very satisfactory feature is the large amount subscribed by the patients themselves and by means of contributory schemes. The 'mass contribution' has been called in to redress the balance of the old individual donor, and it has risen to the occasion."

### Reduction in Expenditure.

Notwithstanding the continuance of the financial depression throughout the country in 1922, the net increase in the ordinary income of the hospitals of £68,578 over the figures for 1921 indicates, as Sir Napier points out, a deepening interest of the public in the Voluntary Hospital Movement. "The remarkable reduction in ordinary expenditure of £275,582—a reduction in which the majority of the hospitals participated—was in large measure accounted for by a fall of approximately 16 per cent. in the expenditure on 'Provisions,' 9 per cent. on 'Surgery and Dispensary,' and 4 per cent. on 'Domestic.' On the other hand, there was a slight increase of some 5 per cent. under the heading of 'Salaries and Wages.'" The hospitals in Scotland apparently receive a richer flow of legacies than those in England, the percentage of ordinary income in Scotland being 21 per cent. as against 15 per cent. in England.

### How to Present Hospital Statistics.

The report notes a marked improvement in the form of presenting hospital statistics, and this has been particularly noticeable in the case of the smaller hospitals :—

"Hospital statistics are of two separate and distinct kinds : Household or domestic statistics, such as the finances of the institution, departmental expenditure, and the personnel employed, etc. ; vital statistics—relating to the patients treated, their number, percentage of available beds occupied during the year, the average length of stay, the nature of the diseases treated, number of operations, etc., together with the statistics of work done in the various departments. The object in the publication of the first group is to aid in the improvement of hospital administration by comparison with other comparable hospitals, whereas the so-called 'Vital statistics' when carefully recorded must lead to the advancement of medical and surgical knowledge. Hospitals could play an even greater part than they do now, in the advancement of medical and surgical science by the adoption of some simplified form of clinical records. The advancement of clinical medicine and surgery is in no small measure dependent on case records, showing, it may be, the results of some special form of treatment, the incidence of certain diseases, etc. The form of case records, of course, is entirely under the control of the honorary medical staff of a hospital, but hospitals could receive valuable guidance in the adoption of a uniform minimum standard of records to be kept, if such standard were to be drawn up by some recognised authority, such as a Joint Committee set up by the Royal Colleges of Physicians and Surgeons."

### Offensive and Defensive Medicine.

Sir Napier goes on to deal with research work :—

"The great work of healing the sick and mending the broken that constitutes the daily practice of an army of voluntary doctors, may be regarded as defensive medicine ; the enemy of disease has wrought his work, and the patient is admitted to hospital in order to have the damage repaired. But another important function of the hospital is that carried on in the hospital laboratories, where investigators are daily at work in seeking to discover the cause and nature of disease. In this work medical science takes the offensive against the enemy. Hospitals rarely get the credit that is their due for the important results attained in the form of new discoveries that add to our knowledge of the prevention of disease. It is even yet too little recognised by the public that the great and ever expanding science of preventive medicine owes its existence in large measure to the work carried out in the hospital laboratories.

### The Small Hospital and the Laboratory.

The equipment of a laboratory is, however, exceedingly costly, and requires patient consideration :—

"In the case of the large hospitals, the laboratory accommodation has evolved during the past twenty-five years from a single room with one trained worker, until now there is a pathological and bacteriological institute attached to many of the hospitals, replete in its many departments and staffed by highly trained personnel, each a specialist in his own particular subject. In the case of the smaller hospitals, the gratifying information is to be found in many annual reports that during the past year steps have been taken to establish some form of laboratory accommodation, and quite a number of hospitals for the first time publish details of laboratory work. One has been invited during the year by a number of such hospitals to discuss with them the difficulties pertaining to the setting up of laboratory facilities requisite for the investigation of the nature of disease. In the case of some of the smaller-sized hospitals, co-operative arrangements have been arrived at between a number of neighbouring hospitals, so that the services of a pathologist may be available for a group of hospitals. In other instances, one has suggested, in solution of this problem, that applicants for the post of resident medical officers should produce evidence of special training and equipment in pathological and bacteriological work, sufficient to enable them to undertake a certain standard of laboratory work.

### Hospital Feeding.

With regard to the feeding of patients, Sir Napier has some cogent things to say. "One of the most important factors in treatment, it has not hitherto received that amount of attention which its importance demands." This portion of the report needs taking to heart by hospital authorities :—

"In a number of hospitals that have been personally visited during the past two years, it was noted that the medical staffs were devoting considerable attention to this question of feeding, and had insisted on the establishment of a special kitchen committee in their hospital with the object of raising the standard of feeding in the hospital. In the domain of surgery the utmost care is lavished upon technique, appliances, instruments, dressings, etc., the quality of the lint, gauze or wool is carefully considered by the surgeon. With the physician, similar care is exercised in the domain of drugs; not only must they conform to a standard of purity that might almost be called absolute, but they are administered in quantities that call for great accuracy in measurement. Nothing is left to chance, all is carefully watched and recorded. Surely the quantity, and the quality, and the presentation of food is of very great importance just as is the dosage and quality of drugs or the variety of surgical dressings."

### A Kitchen Committee.

Sir Napier insists that the monotony of institution feeding cannot but react unfavourably upon the patient :—

"It would be a considerable advance if more hospitals adopted the idea of a kitchen committee that would go into such matters as quality of provisions bought, the equipment and staff of the hospital kitchen, the preparation of food, and especially its presentation to the patient. Such a committee should be a standing committee with regular work to do, not merely called together when complaints are made, and it is necessary to defend the hospital in the eyes of the public. Again, it would be of great advantage were each of the large hospitals to have a trained dietitian who would assist the doctor and work out for him a diet suitable to the complaint of the individual patient. It is not suggested that this would be necessary in the case of all patients, for after all, the bulk of hopsital patients require nothing more than good plain food, well cooked, and appetisingly served. Increased attention, as above outlined, to the question of patient feeding, will probably raise the cost per bed, but any increase in the cost of 'Provisions' may be counterbalanced by a reduction on 'Drugs.' However, the public will never fail to respond to the call for additional money required in the improvement of the feeding of hospital patients."

### Empty Beds in Cottage Hospitals.

Sir Arthur Stanley justly claims consideration for the figures given by Sir Napier with reference to cottage hospitals. In the cottage hospitals of England and Scotland, on the average approximately 2,000 beds out of a total of 5,179 remained idle throughout 1922. Two suggestions are made whereby beds in these hospitals might become of more use to the community :—

"Many of these cottage hospitals could negotiate with the large hospitals in neighbouring towns to take in some patients in an early state of their convalescence, and thus enable these overpressed town hospitals to overtake their waiting lists of patients. Many cottage hospitals with empty beds could render excellent service to the local communities in which they are situate by making arrangements to receive maternity cases, especially first cases, from the local villages around. Institutional provision for such work in rural areas is extremely limited; even the supply of trained maternity nurses is very inadequate. Surely in agreeing to undertake this work the cottage hospital might render valuable assistance in reducing the present high rate of maternal mortality and morbidity."

Well may Sir Arthur commend this section of the report, not only to those in charge of cottage hospitals, but also to the governors of hospitals in crowded districts which are always faced with a long list of patients awaiting admission.

### Four Years of Progress.

The establishment by the Joint Council of an Information Bureau has met with great encouragement. It has been utilised for hospital information during the past year by Government Departments (War, India, and Colonial Offices), by several of the Voluntary Hospital Committees, and also by the Governments of several of the Overseas Dominions. "The use that is already being made of this clearing house of information," writes Sir Arthur Stanley, "warrants the assertion that Sir Napier Burnett's Annual Report—and even more the vast amount of work which it entails—has contributed in no small degree to the wonderful progress which Voluntary Hospitals have made since they were relieved from the stress of war only a short four years ago."

---

### A HOSPITAL TELEPHONE.

THE "Relay" Automatic Telephone System—the latest development in automatic telephony —is of unusual interest in that it altogether eliminates the electro-mechanical switchboard. There are in the "Relay" switchboards no parts that can wear out and require skilled attention, the whole of the operations are controlled and actuated electrically, the only moving parts in the installation being the "Relay" armatures, which require neither oil nor adjustment. All the operations of number-selecting, connecting and disconnecting are performed by telephone relay, and in this way one department is able to get into touch with another without the intervention of the human operator. The "Relay" system would therefore seem to be the ideal installation for any large building, and its adoption by the Governments of Great Britain, India, Australasia and Sweden, and by the leading business houses, proves that it is thoroughly to be depended upon.

### Simplicity and Economy.

Simple, since it requires no skilled attention, and economical, since it requires no operator and gets a connection in four seconds, the system seems to us to be peculiarly suitable for installation in the larger hospitals. It has, indeed, already been adopted by the London Hospital, the Royal Northern Hospital, the South London Hospital for Women, and several smaller institutions, while the Secretary of the Chelsea Hospital for Women writes that the system has been in use there during the last seven years. "It has proved itself invaluable as a means of communication between its different departments at all hours of the day and night. No operator is required, communications are readily made and are entirely secret." The Middlesex Hospital and the London Temperance Hospital are now having the "Relay" installed, and the company at Marconi House, Strand, will on application be glad to send a booklet which describes more minutely than we can do here the working of this excellent system in hospitals.

